# Optogenetic inhibition of D1R containing nucleus accumbens neurons alters cocaine-mediated regulation of Tiam1

**DOI:** 10.3389/fnmol.2013.00013

**Published:** 2013-05-24

**Authors:** Ramesh Chandra, Jeffrey D. Lenz, Amy M. Gancarz, Dipesh Chaudhury, Gabrielle L. Schroeder, Ming-Hu Han, Joseph F. Cheer, David M. Dietz, Mary Kay Lobo

**Affiliations:** ^1^Department of Anatomy and Neurobiology, University of Maryland School of MedicineBaltimore, MD, USA; ^2^Department of Pharmacology and Toxicology, The State University of New York at BuffaloBuffalo, NY, USA; ^3^The Research Institute on Addictions, The State University of New York at BuffaloBuffalo, NY, USA; ^4^Department of Pharmacology and Systems Therapeutics, Friedman Brain Institute, Mount Sinai School of MedicineNew York, NY, USA

**Keywords:** optogenetics, Tiam1, nucleus accumbens, medium spiny neurons, cocaine

## Abstract

Exposure to psychostimulants results in structural and synaptic plasticity in striatal medium spiny neurons (MSNs). These cellular adaptations arise from alterations in genes that are highly implicated in the rearrangement of the actin-cytoskeleton, such as T-lymphoma invasion and metastasis 1 (Tiam1). Previous studies have demonstrated a crucial role for dopamine receptor 1 (D1)-containing striatal MSNs in mediating psychostimulant induced plasticity changes. These D1-MSNs in the nucleus accumbens (NAc) positively regulate drug seeking, reward, and locomotor behavioral effects as well as the morphological adaptations of psychostimulant drugs. Here, we demonstrate that rats that actively self-administer cocaine display reduced levels of Tiam1 in the NAc. To further examine the cell type-specific contribution to these changes in Tiam1 we used optogenetics to selectively manipulate NAc D1-MSNs or dopamine receptor 2 (D2) expressing MSNs. We find that repeated channelrhodopsin-2 activation of D1-MSNs but not D2-MSNs caused a down-regulation of Tiam1 levels similar to the effects of cocaine. Further, activation of D2-MSNs, which caused a late blunted cocaine-mediated locomotor behavioral response, did not alter Tiam1 levels. We then examined the contribution of D1-MSNs to the cocaine-mediated decrease of Tiam1. Using the light activated chloride pump, eNpHR3.0 (enhanced *Natronomonas pharaonis* halorhodopsin 3.0), we selectively inhibited D1-MSNs during cocaine exposure, which resulted in a behavioral blockade of cocaine-induced locomotor sensitization. Moreover, inhibiting these NAc D1-MSNs during cocaine exposure reversed the down-regulation of Tiam1 gene expression and protein levels. These data demonstrate that altering activity in specific neural circuits with optogenetics can impact the underlying molecular substrates of psychostimulant-mediated behavior and function.

## INTRODUCTION

Striatal medium spiny neurons (MSNs) play a crucial role in the molecular, cellular, and behavioral responses to psychostimulants (Lee et al., 2006; Dietz et al., 2009, 2012; Hikida et al., 2010; Lobo et al., 2010; Ferguson et al., 2011; Kim et al., 2011; Lobo and Nestler, 2011; Robison and Nestler, 2011). Previous studies demonstrate a role for dopamine receptor 1 (D1) containing striatal MSNs to positively regulate psychostimulant induced molecular, behavioral, and cellular plasticity adaptations in the striatum whereas dopamine receptor 2 (D2) expressing MSNs negatively regulate psychostimulant-mediated behaviors (Moratalla et al., 1992; Lee et al., 2006; Bertran-Gonzalez et al., 2008; Hikida et al., 2010; Lobo et al., 2010; Ferguson et al., 2011; Kim et al., 2011; Lobo and Nestler, 2011; Pascoli et al., 2011; Grueter et al., 2013). Consistent with these findings, cocaine-induced structural plasticity and synaptic plasticity alterations in the nucleus accumbens (NAc) predominantly occur in D1-MSNs (Lee et al., 2006; Kim et al., 2011; Pascoli et al., 2011; Grueter et al., 2013). These adaptations occur in both the dorsal striatum and NAc, two key areas of the “reward” circuitry. Both brain regions are part of the striatum; the NAc along with the olfactory tubercle composes the ventral striatum (Ikemoto, 2007). Exposure to cocaine or other psychostimulants alters the expression of dendritic plasticity, spine growth, and actin remodeling associated genes that regulate these structural plasticity changes in NAc MSNs (Toda et al., 2006; Pulipparacharuvil et al., 2008; Dietz et al., 2009, 2012; Kalivas, 2009). Conversely, disrupting these molecules can impact cocaine-induced structural plasticity and behaviors (Toda et al., 2006; Pulipparacharuvil et al., 2008; Dietz et al., 2009, 2012; Kalivas, 2009). It is unknown if these molecular changes are mediated in a cell type-specific manner.

One such set of molecules altered by cocaine are cytoskeletal-associated proteins that can directly alter structural plasticity, including spine density and dendritic branching in MSNs (Robinson and Kolb, 2004; Dietz et al., 2009, 2012; Kalivas, 2009; Russo et al., 2010; Kim et al., 2011). The actin-cytoskeleton is primarily regulated by the Rho family small GTPase molecules (Schubert and Dotti, 2007; Cingolani and Goda, 2008). Recently, we demonstrated that Rac1 (Ras-related C3 botulinum toxin substrate 1), a small GTPase that controls actin remodeling, is negatively regulated after repeated cocaine exposure, and enhanced Rac1 signaling blocks cocaine-mediated structural and behavioral plasticity (Dietz et al., 2012). The cocaine-induced decrease in Rac1 activity was accompanied by down-regulation in T-lymphoma invasion and metastasis 1 (Tiam1) protein, the upstream regulator of Rac1 (Dietz et al., 2012) and a decrease in Tiam1 gene expression (unpublished data). Tiam1 is highly expressed in spines and dendrites and plays an important role in dendritic structure and synaptic plasticity (Tolias et al., 2007).

Our previous study examined Tiam1 levels in the NAc after non-contingent home cage cocaine i.p. injections (Dietz et al., 2012) but did not assess whether Tiam1 was regulated in more behaviorally relevant models of addiction (i.e., self-administration, behavioral sensitization). We first investigated if Tiam1 expression in MSNs was changed following cocaine self-administration. We next examined whether channelrhodopsin-2 (ChR2) optogenetic activation of D1-MSNs or D2-MSNs could alter Tiam1 levels. To further understand the contribution of D1-MSN cell type specificity in the regulation of Tiam1 following cocaine exposure, we use optogenetics to selectively inhibit D1-MSNs. We used enhanced *Natronomonas pharaonis* halorhodopsin 3.0 (eNpHR3.0), the yellow/green light activated chloride pump, during cocaine-induced locomotor sensitization and then examined Tiam1 gene expression and protein levels. Overall our studies demonstrate the regulation of Tiam1 following behavioral sensitization and cocaine self-administration. Moreover, we now begin to examine how cell type specificity, particularly the D1-MSNs, contributes to the underlying molecular mechanisms of psychostimulant induced structural plasticity and behavior. Furthermore, we examine whether altering activity in specific circuits with optogenetics can influence these underlying molecular mechanisms.

## MATERIALS AND METHODS

### ANIMALS

Mice used for optogenetic experiments were D1-Cre hemizygote (line FK150) or D2-Cre hemizygote (line ER44) bacterial artificial chromosome (BAC) transgenic mice on a C57Bl/6 background (Gong et al., 2007, genstat.org). All mice were maintained on a 12-h light/dark cycle *ad libitum* food and water. Sprague-Dawley rats, used in the self-administration experiment, were maintained on a 12-h reverse light/dark cycle *ad libitum* food and water. All studies were conducted in accordance with the guidelines set up by the Institutional Animal Care and Use Committee’s at The University of Maryland School of Medicine, The University at Buffalo, The State University of New York, and Mount Sinai Medical School.

### *IN VITRO* PATCH-CLAMP ELECTROPHYSIOLOGY

Whole-cell recordings were obtained from NAc MSNs in acute brain slices from D1-Cre mice that were injected with adeno-associated virus (AAV)-double-floxed inverted orientation (DIO)-eNpHR3.0-enhanced yellow fluorescent protein (EYFP; AAV-DIO-eNpHR3.0-EYFP; Gradinaru et al., 2010; Witten et al., 2010; Stuber et al., 2011; Tye et al., 2011; Chaudhury et al., 2013; Stefanik et al., 2013) into the NAc. To minimize stress and to obtain healthy NAc slices, mice were anesthetized immediately when they were brought to electrophysiology area and perfused for 40–60 s with ice-cold aCSF (artificial cerebrospinal fluid), which contained 128 mM NaCl, 3 mM KCl, 1.25 mM NaH_2_PO_4_, 10 mM D-glucose, 24 mM NaHCO_3_, 2 mM CaCl_2_, and 2 mM MgCl_2_ (oxygenated with 95% O_2_ and 5% CO_2_, pH 7.4, 295–305 mOsm). Acute brain slices containing NAc were cut using a microslicer (Ted Pella) in cold sucrose-aCSF, which was derived by fully replacing NaCl with 254 mM sucrose and saturated by 95% O_2_ and 5% CO_2_. Slices were maintained in holding chamber with aCSF for 1 h at 37°C. Patch pipettes (3–5 MΩ), for whole-cell current-clamp and voltage-clamp recordings, were filled with internal solution containing the following: 115 mM potassium gluconate, 20 mM KCl, 1.5 mM MgCl_2_, 10 mM phosphocreatine, 10 mM HEPES [4-(2-hydroxyethyl)-1-piperazineethanesulfonic acid], 2 mM magnesium ATP, and 0.5 mM GTP (pH 7.2, 285 mOsm). Whole-cell recordings were carried out using aCSF at 34°C (flow rate = 2.5 ml/min). For current-clamp recordings to measure green (532 nm) light inhibition of action potentials we measured the effect of green light eNpHR3.0 activation on current (50 pA) injection-induced spiking. eNpHR3.0 activation-induced outward current was measured in voltage-clamp. Current- and voltage-clamp experiments were performed using the Multiclamp 700B amplifier and data acquisition was carried out in pClamp 10 (Molecular Devices). Series resistance was monitored during the experiments, and membrane currents and voltages were filtered at 3 KHz (Bessel filter). For light delivery to NAc slices, a 200 μm optical fiber (Thor Labs, BFL37-200) was connected via a FC (fiber-optic)/PC (physical contact) adaptor to a 532 nm green laser diode (OEM Laser Systems).

### IMMUNOHISTOCHEMISTRY

D1-Cre mice were perfused with 0.1 M phosphate buffered saline (PBS) followed by 4% paraformaldehyde (PFA). Brains were immersed in PFA overnight and then cryopreserved in 30% sucrose. Brains were cryosectioned (Leica) at 35 μm into 0.1 M PBS. Brain sections were blocked in 3% normal donkey serum with 0.3% Triton-X for 1 h at room temperature. Sections were then incubated overnight at room temperature in primary antibodies, 1:5,000 chicken anti-GFP (Aves) and 1:1,000 mouse anti-Cre (Millipore) diluted in the above blocking solution. On the second day, tissue sections were rinsed in 0.1 M PBS followed by a 1-h incubation at room temperature in secondary antibodies, 1:1,000 donkey anti-chicken-Alexa488 and 1:1,000 donkey anti-mouse-Cy3 (Jackson ImmunoResearch). Immunofluorescence was imaged on an Olympus Bx61 confocal microscope.

### COCAINE SELF-ADMINISTRATION

For self-administration (SA) studies, male Sprague-Dawley rats weighing approximately 300 g at the start of the experiments were used. Rats were implanted with chronic indwelling jugular catheters and singly housed for the rest of the SA phase of the experiment in order to protect the catheter/harness assembly. The catheter implantations, maintenance, and patency testing procedures have been previously described elsewhere (Gancarz et al., 2012). Only rats with patent catheters at the end of the study were used in data analysis.

One week after jugular catheter surgery, the rats assigned to self-administer saline (*n* = 6) or 1.0 mg/kg/infusion cocaine (*n* = 6). Cocaine hydrochloride (National Institute on Drug Abuse) was dissolved in sterile saline. Two-hour daily testing occurred during the animals” dark cycle in Med-Associates experimental test chambers fitted with two snout poke ports, using a fixed ratio (FR) 1 schedule for 10 test sessions (Renthal et al., 2009; Gancarz et al., 2012). Twenty-four hours following the last self-administration session, brains were harvested, and NAc tissue punches were quickly collected and stored at -80°C.

### MOUSE STEREOTAXIC SURGERY

D1-Cre or D2-Cre mice were anesthetized using 4% isoflurane in a small induction chamber. After the initial induction, isoflurane was maintained at 1% for the remainder of the surgery. Animals were placed in a stereotaxic instrument, and skull exposed. Thirty-three gage Hamilton syringe needles were used to inject 0.6 μl of either AAV-DIO-eNpHR3.0-EYFP, AAV-DIO-ChR2-EYFP, or AAV-DIO-EYFP bilaterally into the NAc (anterior/posterior, AP +1.6; medial/lateral, ML ±1.5; dorsal/ventral, DV -4.4, 10° angle). After injections, implantable fibers (Sparta et al., 2011; 4.0 mm in length, 105 μm core) were lowered into the NAc (AP +1.6, ML ±1.5, DV -4.0, 10° angle). Instant adhesive was used to fix the implantable fiber to the skull, followed by dental cement to anchor the fibers. Animals were then returned to the vivarium for 2 weeks to allow for recovery and maximal virus expression.

### MOUSE COCAINE LOCOMOTOR ACTIVITY AND LIGHT STIMULATION

D1-Cre or D2-Cre mice expressing either AAV-DIO-eNpHR3.0-EYFP, AAV-DIO-ChR2-EYFP, or AAV-DIO-EYFP in NAc D1-MSNs or D2-MSNs underwent 3 days of 60-min habituation sessions in a 42 cm × 42 cm × 42 cm open field chamber after receiving saline i.p. injections. On the final 2 days of habituation, a 62.5-μm split fiber patch cord was attached to the implantable head mount fibers before animals were placed into the chamber. The animals then underwent five daily 60-min test sessions where animals were given either 10 mg/kg cocaine i.p. injections or saline 1 ml/kg i.p. injections coupled with continuous green light illumination or 10 Hz blue light illumination (for 30 s every minute; 3.0 mW at the tip) bilaterally to the NAc through a 62.5-μm split fiber patch cord coupled to a green (532 nm) or blue (473 nm) laser diode (OEM) by FC/PC connection. Locomotion was video tracked with Topscan tracking software (Clever Sys, Inc.). The animals were then sacrificed 24 h after the final session and NAc punches were quickly collected and stored at -80°C.

For ChR2 stimulation to D1-MSNs and D2-MSNs for RT^2^ PCR Profiler Array experiments, blue light stimulation was performed in the home cage. Mice received 15 min of 10 Hz blue (473 nm) light pulses (for 30 s every minute) for 5 days. The animals were then sacrificed 24 h after the final session and NAc punches were quickly collected and stored at -80°C.

### RNA EXTRACTION AND QUANTITATIVE RT-PCR

All NAc tissue punches were collected 24 h after the last cocaine administration and stored at -80°C. RNA was extracted using Trizol (Invitrogen) and the RNeasy Micro Kit (Qiagen) with a DNase step. RNA concentration was measured by Nanodrop Spectrophotometer and 400 ng cDNA was then synthesized using reverse transcriptase iScript cDNA synthesis kit (Bio-Rad). mRNA expression changes were measured using quantitative polymerase chain reaction (qPCR) with SsoAdvanced SYBR Green Supermix kit (Bio-Rad) or IQ SYBR Green Supermix (Bio-Rad). Quantification of mRNA changes was performed using the delta delta *C*_T_ method described previously (Maze et al., 2010), using glyceraldehyde 3-phosphate dehydrogenase (GAPDH) as a housekeeping gene. The list of primers used includes:

Tiam1 mouse, forward: CGGAACAGATGGAAACACCT, reverse: AGAGACGACCACCCTTTCCT; Tiam1 rat, forward: GGTGCTGACACACGCCCCAA, reverse: AGGAAGCATGCCGCGTCCTC; caspase 1 mouse, forward: ATGCCTGGTCTTGTGACTTGG, reverse: AATGTCCCGGGAAGAGGTAGA; caspase 3 mouse, forward: AGCTGTCAGGGAGACTCTCAT, reverse: TTGAGGTAGCTGCACTGTGG; caspase 8 mouse, forward: GCGTGAACTATGACGTGAGC, reverse: AAGCCATGTGAACTGTGGAGA; caspase 9 mouse, forward: CGAAGCTCTCATGGCTTGGA, reverse: ACTGCTCCACATTGCCCTAC; neuronal nuclei (NeuN) mouse, forward: CAGACACTGGGGAAGACCTG, reverse: CCTAGGAACCCCCTGTCTGG; GAPDH mouse, forward: AGGTCGGTGTGAACGGATTTG, reverse: TGTAGACCATGTAGTTGAGGTCA; GAPDH rat, forward AACGACCCCTTCATTGAC, reverse TCCACGACATACTCAGCA.

### RT^2^ PCR PROFILER ARRAY

RNA was extracted from ChR2 expressing NAc of D1-Cre or D2-Cre mice as described above and reverse transcribed using RT^2^ First Strand Kit, then run on a RT^2^ PCR Profiler Array (Mouse Cytoskeleton Regulators PCR array, SA Biosciences), according to manufacture’s protocols and as previously described (Lobo et al., 2010). Δ*C*_T_ was determined using SABiosciences software and then normalized to D1-Cre or D2-Cre AAV-DIO-EYFP control treated NAc tissue to obtain 2^-ΔΔCt^ values. Heat maps were generated using Java Tree software and expressed in log scale.

### WESTERN BLOTS

All NAc tissue punches were collected 24 h after the last cocaine administration and stored at -80°C. NAc punches were homogenized in 30 μl of lysis buffer containing 320 mM sucrose, 5 nM HEPES buffer, 1% sodium dodecyl sulfate (SDS), phosphatase inhibitor cocktails I and II (Sigma, St. Louis) and protease inhibitors (Roche) using a ultrasonic processor (Cole Parmer, Vernon Hills, IL, USA). Protein concentrations were determined using DC protein assay (Bio-Rad) and then 20 μg samples of total protein were loaded onto a 7.5% precast Tris–HCl polyacrylamide gel (Bio-Rad). The samples were transferred to a nitrocellulose membrane and blocked for 1 h in blocking buffer, 5% non-fat dry milk in Tris buffered saline (pH 7.6) with 0.1% Tween. Blocked membranes were incubated overnight at 4°C in blocking buffer with primary antibodies using either 1:1,000 rabbit-anti-Tiam1 (Millipore, cat. # ST1070) or 1:2,000 β-Tubulin (Cell Signaling, cat. # 2128S). Membranes were then incubated with goat anti-rabbit peroxidase-labeled secondary antibodies (Vector Laboratories, cat.# PI-1000, 1:20,000 or 1:40,000 depending on the primary antibody used) in blocking buffer. The bands were visualized using SuperSignal West Dura Extended Duration substrate (Pierce). Bands were quantified with ImageJ Software and normalized to β-Tubulin to control for equal loading.

### STATISTICAL TESTS

Statistical analysis was performed with Graphpad Prism software. Repeated measures. Two-way analysis of variance (ANOVA) followed by with Bonferroni *post hoc* test were used to analyze the self-administration and cocaine-mediated locomotor activity data. Student’s *t*-test or two-way ANOVAs followed by a Tukey’s *post hoc* test were used for mRNA, RT^2^ PCR Profiler Array, and protein analysis.

## RESULTS

**FIGURE 1:**
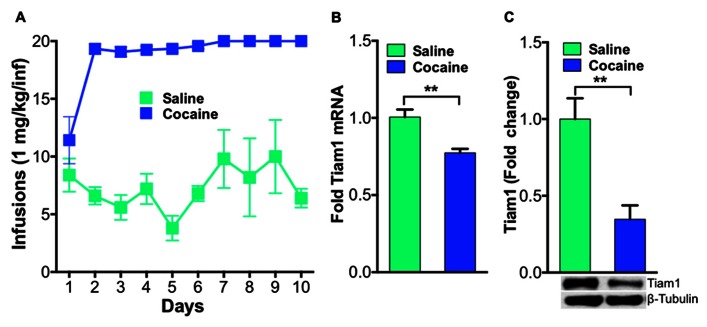
**Tiam1 is decreased in the NAc of rats that self-administer cocaine.(A)** Self-administration of cocaine (1.0 mg/kg/infusion, FR1 schedule) or saline over a 10-day period. Rats self-administering cocaine took more infusions than saline controls on days 2–10 of testing (*n* = 6 per group, two-way repeated measures ANOVA, *F*(9,90) = 4.139, *p* < 0.0001, *post hoc* test, *p* < 0.05). **(B)** Following cocaine self-administration, Tiam1 mRNA is significantly decreased in the NAc compared to saline controls (*n* = 6 per group, Student”s *t*-test, **p < 0.01). **(C)** Tiam1 protein levels are similarly down-regulated in the NAc in rats that self-administer cocaine compared to saline controls (*n* = 4–7 per group, Student”s *t*-test, **p < 0.01).

Previously, we demonstrated that Tiam1 protein levels were down-regulated after repeated home cage cocaine exposure (Dietz et al., 2012) and also found this was accompanied by decreased Tiam1 gene expression (unpublished data). To expand these findings and determine if Tiam1 levels are similarly regulated in the NAc, in rodent models of addiction, we first examined Tiam1 gene expression and protein levels in NAc after contingent cocaine administration conditions in rats. We collected NAc from rats that were trained to self-administer cocaine (**Figure 1A**) for RNA and protein isolation. We find Tiam1 gene expression and protein levels are decreased in the NAc of rats that self-administer cocaine (**Figures 1B,C**).

**FIGURE 2:**
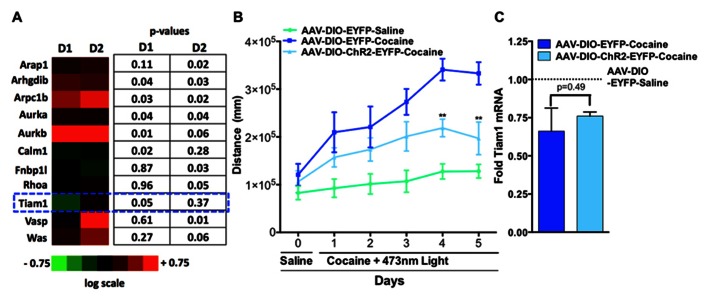
**Optogenetic activation of NAc D1-MSNs but not D2-MSNs alters Tiam1 levels. (A)** D1-Cre or D2-Cre mice expressing AAV-DIO-ChR2-EYFP or AAV-DIO-EYFP in NAc received 5 days of blue light illumination to the NAc (*n* = 4 per group) and NAc tissue was used to perform gene expression analyzes with the Mouse Cytoskeleton Regulators PCR array (SA Biosciences). We find that three genes were significantly regulated in D1-ChR2 NAc compared to D1-EYFP NAc, four genes were significantly regulated in D2-ChR2 NAc compared to D2-EYFP, and three genes were significantly regulated in both D1-ChR2 and D2-ChR2 compared to EYFP controls. Tiam1 was a gene that was significantly down-regulated by repeated D1-ChR2 activation but not by D2-ChR2 activation. Heat maps are expressed in log scale. **(B)** D2-Cre mice expressing AAV-DIO-ChR2-EYFP or AAV-DIO-EYFP in NAc were injected (i.p.) with saline (day 0) followed by injections (i.p.) with either cocaine (10 mg/kg) or saline coupled with blue light illumination to the NAc and locomotor activity was monitored for 1 h (days 1–5, *n* = 5–6 per group). D2-MSN activation in the AAV-DIO-ChR2-EYFP cocaine group resulted in a significant attenuation in cocaine locomotor activity on days 4 and 5 compared to the AAV-DIO-EYFP cocaine group. [Repeated measure two-way ANOVA, *F*(10, 70) = 7.46, *p* < 0.001, *post hoc* test, **p < 0.01.] **(C)** No difference is observed in Tiam1 mRNA levels in AAV-DIO-EYFP and AAV-DIO-ChR2-EYFP cocaine groups (*n* = 5–6).

Since altering activity in D1-MSNs vs. D2-MSNs oppositely regulates psychostimulant-mediated behaviors and reinforcement (Lobo et al., 2010; Ferguson et al., 2011; Kravitz et al., 2012) we next tested whether repeated blue light activation of ChR2 expressing D1-MSNs or D2-MSNs *in vivo* in the NAc could alter cytoskeleton regulated genes. We expressed ChR2 or EYFP in the NAc of D1-MSNs and D2-MSNs by stereotaxic injection of the Cre-inducible adeno-associated virus AAV-DIO-ChR2-EYFP or AAV-DIO-EYFP to the NAc in D1-Cre and D2-Cre BAC transgenic mice. Mice received 5 days of 15 min blue light (473 nm) pulses to the NAc and gene expression analysis was performed on NAc tissue (Lobo et al., 2010) using the SABiosciences Mouse Cytoskeleton Regulators PCR array. We find that three genes were significantly regulated in D1-ChR2 NAc compared to D1-EYFP NAc, four genes were significantly regulated in D2-ChR2 NAc compared to D2-EYFP, and three genes were significantly regulated in both D1-ChR2 and D2-ChR2 compared to EYFP controls. Tiam1 was a gene that was significantly down-regulated by repeated D1-ChR2 activation, similar to the effects of repeated cocaine exposure, but not by D2-ChR2 activation (**Figure 2A**). Similarly, we see no change in Tiam1 mRNA levels after optogenetic activation of D2-MSNs during cocaine exposure, compared to EYFP cocaine controls despite observing decreased cocaine-mediated locomotor activity on days 4 and 5 under these conditions (**Figures 2B,C**).

**FIGURE 3:**
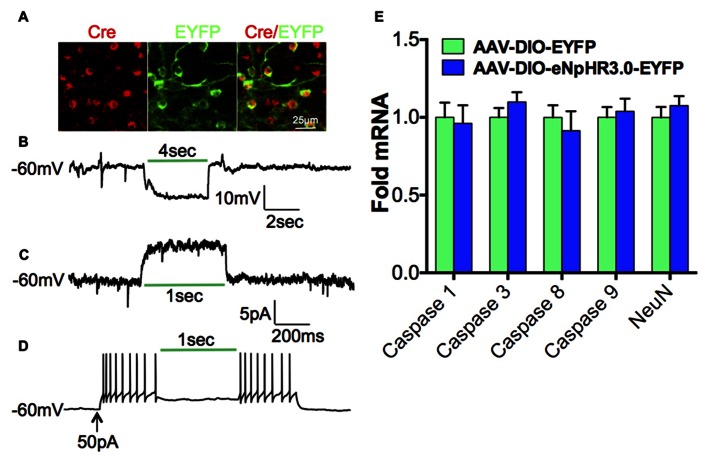
**Optogenetic inhibition of D1-MSNs.(A)** Immunofluorescence image of D1-Cre NAc expressing eNpHR3.0-EYFP (green) in Cre positive D1-MSNs (red; scale bar, 25 μm). (B) Hyperpolarization and (C)outward photo-current of the membrane potential in eNpHR3.0 expressing D1-MSNs during green (532 nm) light illumination. **(D)** Inhibition of neuronal firing during green (532 nm) light illumination in NAc D1-MSNs expressing eNpHR3.0. **(E)** 5-day, 60-min per day green light illumination (paradigm used in **Figure 4**) to the NAc does not alter cell death markers or NeuN gene expression in D1-Cre NAc expressing AAV-DIO-eNpHR3.0-EYFP or AAV-DIO-EYFP (Student”s *t*-test, *p* > 0.05).

We next investigated the contribution of D1-MSNs to the cocaine-mediated decrease in Tiam1, since many studies have demonstrated a positive role for D1-MSNs in the dorsal striatum and NAc in mediating the molecular, cellular, and behavioral responses to drugs of abuse (Lobo and Nestler, 2011). To assess the role of D1-MSN cell type specificity in mediating the cocaine-induced Tiam1 reduction, we expressed the yellow/green light activated chloride pump, eNpHR3.0 (Gradinaru et al., 2010; Witten et al., 2010; Stuber et al., 2011; Tye et al., 2011; Chaudhury et al., 2013; Stefanik et al., 2013) in the NAc by stereotaxic injection of the Cre-inducible AAV-DIO-eNpHR3.0-EYFP or AAV-DIO-EYFP in D1-Cre BAC transgenic mice (**Figure 3A**). We first validated the utility of green light (532 nm) to photo-inhibit NAc D1-MSNs expressing eNpHR3.0 and observed hyperpolarization of the membrane potential as well as an outward current during green light illumination of eNpHR3.0 expressing D1-MSNs (**Figures 3B,C**). As expected, green light delivered to NAc D1-MSNs expressing eNpHR3.0 caused a decrease in D1-MSN firing (**Figure 3D**). Finally, we found that 5 days of 60-min green light illumination to the NAc *in vivo* does not alter markers of cell death or NeuN gene expression (**Figure 3E**) in NAc of D1-Cre mice expressing EYFP or eNpHR3.0-EYFP in D1-MSNs.

**FIGURE 4:**
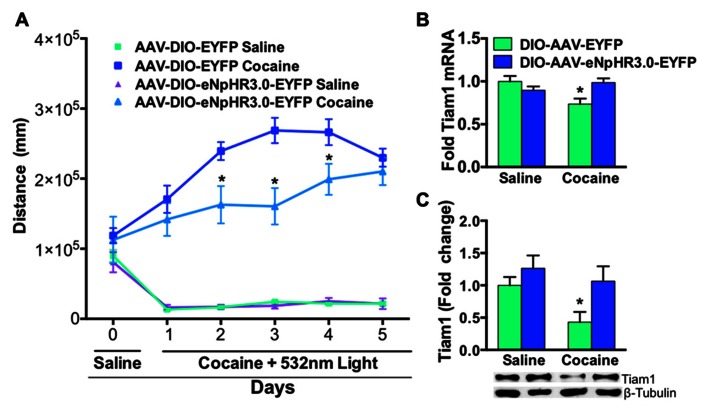
**Optogenetic inhibition of NAc D1-MSNs attenuates cocaine locomotor sensitization and reverses Tiam1 gene expression. (A)** D1-Cre mice expressing AAV-DIO-eNpHR3.0-EYFP or AAV-DIO-EYFP in NAc were injected (i.p.) with saline (day 0) followed by injections (i.p.) with either cocaine (10 mg/kg) or saline coupled with green light illumination to the NAc and locomotor activity was monitored for 1 h (days 1–5, *n* = 4–7 per group). D1-MSN inhibition in the AAV-DIO-eNpHR3.0-EYFP cocaine group resulted in a significant attenuation in cocaine locomotor activity on days 2–4 compared to the AAV-DIO-EYFP cocaine group. (Repeated measure two-way ANOVA, *F*(15,85) = 13.03, *p* < 0.0001, *post hoc* test, *p < 0.05.) **(B,C)** Tiam1 mRNA and protein is significantly down-regulated in the NAc of the cocaine AAV-DIO-EYFP group compared to the saline AAV-DIO-EYFP and AAV-DIO-eNpHR3.0-EYFP saline groups. Inhibition of D1-MSNs in the AAV-DIO-eNpHR3.0 cocaine group reversed the blunted Tiam1 cocaine response (mRNA: *n* = 4–7 per group, two-way ANOVA, interaction, *F*(1,16) = 7.64, *p* < 0.05, *post hoc* test, *p < 0.05; protein: *n* = 4–7 per group, two-way ANOVA, group effect, *F*(1,16) = 6.24, *p* < 0.05, drug effect, *F*(1,16) = 4.60, *p* < 0.05, *post hoc* test, *p < 0.05).

We then illuminated NAc D1-MSNs, expressing eNpHR3.0 or EYFP, *in vivo* with green light during repeated saline or cocaine (10 mg/kg, i.p.) injections over a 5-day period. As expected, cocaine treated D1-Cre mice expressing EYFP displayed enhanced cocaine-induced locomotion when compared to saline treated EYFP or eNpHR3.0 mice (**Figure 4A**). In contrast, inhibition of D1-MSNs, expressing eNpHR3.0, with green light significantly blocked cocaine-induced locomotion (**Figure 4A**). We observed significant differences on days 2–4 in cocaine treated D1-Cre eNpHR3.0 mice relative to D1-Cre EYFP cocaine treated mice (**Figure 4A**). We next tested whether blocking cocaine-induced locomotor activity by silencing D1-MSNs can alter Tiam1 gene expression and protein levels in the NAc. As expected, we found that D1-Cre mice expressing EYFP had reduced levels of Tiam1 gene expression and protein levels in the NAc after 5 days of repeated cocaine (10 mg/kg, i.p.) when compared to the saline EYFP or saline eNpHR3.0 groups (**Figures 4B,C**). Consistent with our findings that inhibiting D1-MSNs, expressing eNpHR3.0, with green light attenuates cocaine-induced behavioral responses; we find that silencing D1-MSNs also reverses the cocaine-mediated negative regulation of Tiam1 gene and protein expression levels (**Figures 4B,C**).

## DISCUSSION

Repetitive cocaine exposure alters structural plasticity changes that are tightly correlated with the induction of behavioral sensitization (Li et al., 2004). These cocaine-induced structural plasticity adaptations are mediated through actin-cytoskeletal proteins, which are crucial for polymerization of actin to alter spine induction, maturation, and stability (Halpain, 2000; Nimchinsky et al., 2002; Toda et al., 2006; Dietz et al., 2009, 2012; Russo et al., 2010). Consistent with previous studies showing a role for cytoskeletal proteins in modulating the behavioral effects of cocaine, we find a potent decrease in the cytoskeletal regulator, Tiam1, after cocaine self-administration and cocaine-induced locomotor sensitization.

The mechanism by which actin-cytoskeleton molecules are regulated after cocaine behavioral plasticity is unknown. We investigated the role for NAc MSN subtypes in regulating the actin-cytoskeleton molecules, including Tiam1, by using optogenetics to control activity in MSN subtypes. We find that increasing neuronal activity repeatedly over 5 days in D1-MSNs resulted in the down-regulation of Tiam1 mRNA, similar to the effects of cocaine. We observe no change in Tiam1 levels when activating D2-MSNs. We then investigated the role for NAc MSN subtypes in regulating the cocaine-mediated decrease in Tiam1 by activating D2-MSNs or inhibiting D1-MSNs during cocaine locomotor behavioral sensitization. Studies from our group and other groups using optogenetics or designer receptor activated by designer drugs (DREADDs) demonstrate a role for dorsal striatum and NAc D1-MSN activity in positively mediating motivational and motor behaviors (Hikida et al., 2010; Kravitz et al., 2010, 2012; Lobo et al., 2010; Ferguson et al., 2011; Tai et al., 2012). Consistent with these studies we demonstrate that optogenetic activation of D2-MSNs or inhibition of D1-MSNs blocks cocaine-induced locomotor sensitization. Interestingly, we observed a blunted cocaine-mediated locomotor response on later days (days 4 and 5) when activating D2-MSNs, whereas cocaine-mediated locomotion was decreased on earlier days (days 2–4) when inhibiting D1-MSNs. Furthermore, we report that repeated optogenetic inhibition of D1-MSNs reverses the cocaine-mediated down-regulation of Tiam1, suggesting that regulation of Tiam1 in D1-MSNs mediates the early behavioral responses to cocaine. These findings reveal evidence for a role of D1-MSN activity in regulating the actin-cytoskeleton after cocaine exposure, consistent with previous studies demonstrating that the structural and synaptic plasticity effects of cocaine occur mainly in D1-MSNs (Lee et al., 2006; Kim et al., 2011; Pascoli et al., 2011; Grueter et al., 2013).

Although the precise molecular mechanisms by which Tiam1 is regulated in NAc MSNs after cocaine exposure is unclear, one intriguing mechanism is through an increase in the transcription factor deltaFosB which may mediate the expression of Tiam1. Indeed, deltaFosB, the long lasting isoform of the FosB gene, is stably increased selectively in D1-MSNs by cocaine (Moratalla et al., 1992; Lee et al., 2006) and has been shown to positively mediate cocaine behavioral and structural plasticity (Kelz et al., 1999; Lee et al., 2006; Maze et al., 2010; Grueter et al., 2013). Future studies examining cocaine-induced deltaFosB or other transcription factor”s regulation of the Tiam1 gene will be crucial to determine the precise molecular mechanisms that mediate Tiam1 expression.

An important caveat to this study is the lack of cell type-specific Tiam1 gene expression or protein expression. Thus, we do not yet know whether the Tiam1 changes are occurring in D1-MSNs or the other MSN subtype or interneurons. Future studies combining these optogenetic manipulations of cocaine-mediated behaviors with cell type selective gene expression profiling (Lobo et al., 2006; Heiman et al., 2008; Guez-Barber et al., 2011) of Tiam1 or other cytoskeleton molecular candidates will be necessary to answer this question. Further, although we demonstrate Tiam1 gene expression and protein levels are decreased in the NAc in rats that actively self-administer cocaine, we do not know if this contingent paradigm causes a cell type-specific regulation of Tiam1 levels. Future studies employing these optogenetic experiments in self-administering (Stefanik et al., 2013) transgenic rodents (Koya et al., 2009; Witten et al., 2011) can provide further insight into these questions.

Collectively, our study associates a role for D1-MSN activity in regulating actin-cytoskeleton dynamics in cocaine dendritic and behavioral plasticity. We furthermore, show the utility of using optogenetics to manipulate psychostimulant-mediated behaviors to define the molecular mechanisms associated with drugs of abuse. Future studies to manipulate activity in specific cell subtypes during drug abuse behavioral paradigms coupled with molecular profiling could potentially lead to novel therapeutic targets to treat drug addiction.

## Conflict of Interest Statement

The authors declare that the research was conducted in the absence of any commercial or financial relationships that could be construed as a potential conflict of interest.
